# Clinical Potential of Himalayan Herb *Bergenia ligulata*: An Evidence-Based Study

**DOI:** 10.3390/molecules27207039

**Published:** 2022-10-18

**Authors:** Shubhadeep Roychoudhury, Dipika Das, Sandipan Das, Niraj Kumar Jha, Mahadeb Pal, Adriana Kolesarova, Kavindra Kumar Kesari, Jogen C. Kalita, Petr Slama

**Affiliations:** 1 Department of Life Science and Bioinformatics, Assam University, Silchar 788011, India; 2 Department of Biotechnology, School of Engineering & Technology (SET), Sharda University, Greater Noida 201310, India; 3 Department of Biotechnology Engineering and Food Technology, Chandigarh University, Mohali 140413, India; 4 Department of Biotechnology, School of Applied & Life Sciences (SALS), Uttaranchal University, Dehradun 248007, India; 5 Division of Molecular Medicine, Bose Institute, Kolkata 700054, India; 6 Faculty of Biotechnology and Food Sciences, Slovak University of Agriculture in Nitra, 94976 Nitra, Slovakia; 7 Department of Bio-products and Bio-systems, School of Chemical Engineering, Aalto University, 00076 Espoo, Finland; 8 Department of Applied Physics, School of Science, Aalto University, 00076 Espoo, Finland; 9 Department of Zoology, Gauhati University, Guwahati 781014, India; 10 Laboratory of Animal Immunology and Biotechnology, Department of Animal Morphology, Physiology and Genetics, Faculty of AgriSciences, Mendel University in Brno, 61300 Brno, Czech Republic

**Keywords:** traditional medicine, *Bergenia ligulata*, bioactive compounds, anti-urolithiatic, antioxidant, anti-pyretic, anti-diabetic, anti-inflammatory, cardiovascular diseases

## Abstract

Herbal products have been used in traditional systems of medicine and by ethnic healers for ages to treat various diseases. Currently, it is estimated that about 80% of people worldwide use herbal traditional medicines against various ailments, partly due to easy accessibility and low cost, and the lower side effects they pose. *Bergenia ligulata*, a herb ranging from the Himalayas to the foothills, including the north-eastern states of India, has traditionally been used as a remedy against various diseases, most prominently kidney stones. The medicinal properties of *B. ligulata* have been attributed to bergenin, its most potent bioactive component. Apart from bergenin, the other compounds available in *B. ligulata* are arbutin, gallic acid, protocatechuic acid, chlorogenic acid, syringic acid, catechin, ferulic acid, afzelechin, paashaanolactone, caryophyllene, 1,8-cineole, β-eudesmol, stigmasterol, β-sitosterol, parasorbic acid, 3-methyl-2-buten-1-ol, phytol, terpinen-4-ol, tannic acid, isovalaric acid, avicularin, quercetin, reynoutrin, and sitoinoside I. This review summarizes various medicinal properties of the herb, along with providing deep insight into its bioactive molecules and their potential roles in the amelioration of human ailments. Additionally, the possible mechanism(s) of action of the herb’s anti-urolithiatic, antioxidative, antipyretic, anti-diabetic, anti-inflammatory and hepatoprotective properties are discussed. This comprehensive documentation will help researchers to better understand the medicinal uses of the herb. Further studies on *B. ligulata* can lead to the discovery of new drug(s) and therapeutics for various ailments.

## 1. Introduction

Traditional herbal medicines are plant-derived natural products that have been used by rural communities for ages for the management of various diseases [1]. Particularly in the tropics and the sub-tropics, an abundance of medicinal plants offers access to effective prevention and management of diseases through self-medication using plant-based medicines. It is estimated that 80% of people worldwide depend on such plant-based traditional medicines for their treatment, and their usage is predominant in developing countries [2]. In recent years, natural products have received renewed global attention from the clinical point of view due to low toxicity, low side effects, cost-effectiveness, and easy accessibility, as compared to modern synthetic medicines [3,4]. *Bergenia ligulata* is a perennial Himalayan herb belonging to the family Saxifragaceae. Another accepted name for the herb is *Bergenia pacumbis*. In the Indian sub-continent, it is distributed along the high-altitude Himalayan regions ranging from Kashmir to Bhutan, including West Bengal and the northeastern states [5,6]. The plant has been used as a folk medicine since ancient times for dissolving kidney stones and is referred to as “*Paashanbheda”* (Sanskrit: *Paashan* meaning “rockstone” and *bheda* meaning “piercing”) in the Indian traditional system of medicine—*Ayurveda* [7]. It has simple leaves of orbicular to obovate shape, stout root stock, and solid barrel-shaped cylindrical rhizome (1.5–3 cm in length and 1–2 cm in diameter) [8]. Himalayan communities consume the roots and rhizomes of the plant to treat wounds, septic, cough and cold, cardiac diseases, asthma, inflammation, gastrointestinal disorders, and different kinds of urinary problems [9]. The most abundant bioactive compound in *B. ligulata* is bergenin [10], which, along with its natural derivatives, mainly contributes to the medicinal properties of the herb [11]. Other important phytochemicals in *B. ligulata* include afzelechin [12], ß-sitosterol [13], catechin, leucocyanidin, gallic acid, and tannic acid [14]. These substances have a number of significant biological activities including anti-bacterial, anti-inflammatory, and free-radical-scavenging properties [9]. An in vivo study on Wistar rats revealed the antilithiatic potential of the plant extract (crude aqueous-methanolic extract) when treated for 21 days at a dose of 5–10 mg/kg body weight, significantly inhibiting calcium oxalate (CaC_2_O_4_) aggregation in the renal ducts [15]. A recent study revealed the ameliorative property of ethanolic extract of *B. ligulata* rhizome against oxalate-mediated renal injury in renal epithelial cells of normal rat kidney 52E (NRK-52E) which was brought about by downregulation of mitogen-activated protein kinases (MAPK), osteopontin (OPN), nuclear factor kappa B (NF-κB), and caspase-3 and reduction of nucleation aggregation and modulation of the crystal structure [16]. Oral administration of the extract, at a dose of 500 mg/kg body weight, in Wistar rats showed strong antipyretic activity against yeast-induced fever [17]. Significant anti-inflammatory and anti-bacterial activities have also been reported after oral administration of 50% ethanolic extract of *B. ligulata* in male Wistar rats at a dose of 1 g/kg body weight [6]. Methanolic extract of *B. ligulata* rhizomes exhibited inhibitory activity against viral RNA and peptide synthesis [18]. In vitro and in vivo studies on *B. ligulata* have shown strong protective properties against *Leishmania donovani* infection, the parasitic load being reduced by >95% at a high dose of 1000 mg/kg body weight in mouse [19]. Notwithstanding its wide-ranging use in traditional medication, scientific evidence on the clinical application of *B. ligulata* remains inadequate. This evidence-based review summarizes the relevant information available on the role of the herb against various kinds of diseases as well as the responsible potent bioactive compounds. The present study also highlights the possible mechanism(s) of action of *B. ligulata* in modulating mammalian physiology.

## 2. Methodology

**Figure 1:**
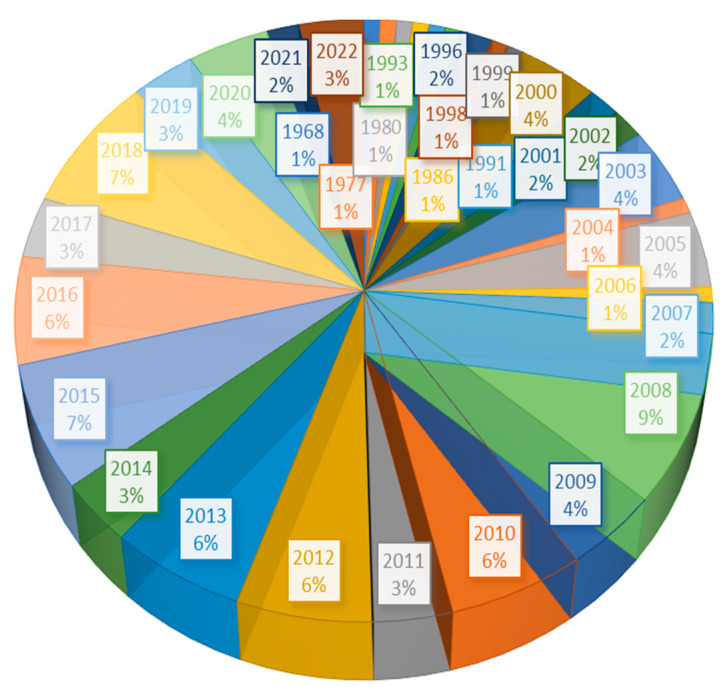
Pie chart illustrating the year-wise distribution of referenced papers.

For the preparation of the present manuscript, the literature regarding the potential clinical use of *B. ligulata* was searched and articles extracted from online databases such as Pubmed, SCOPUS, Google Scholar, and Science Direct. Keyword strings such as (traditional medicine) AND (*Bergenia ligulata* OR *Saxifraga ligulata* Wall OR *Saxifraga thysanodes* OR *Bergenia pacumbis*), (bioactive molecules) AND (*Bergenia ligulata*), (bergenin) AND (anti-lithiatic activity), (catechin), (bergenin) AND (antioxidant), (inflammation), (pyretic), (cardiovascular disease), (diabetes), (hepatoprotective) were used for searching the literature in SCOPUS and Pubmed. After that, only relevant book chapters, full-text articles, and abstracts were screened, and unrelated articles, as well as publications on languages other than English, were excluded from the study as those publications do not relate to the specific aim of the review article. Thus selected articles (Figure 1) were critically analyzed, and results were organized into several sections in the manuscript. Finally, a possible mechanism of action of *B. ligulata* was also speculated as an outcome of the study. At the end of the article future perspective is also considered.

## 3. *Bergenia ligulata* as Traditional Herbal Medicine

A huge recovery in the interest and use of medicinal plants has been witnessed in the previous decade. For a considerably long time, the plants belonging to the genus *Bergenia* have received notable attention for their restorative properties against ailments and have been broadly utilized in traditional medicines in various regions, particularly in the Asian continent, including India, Pakistan and Nepal [20]. Rhizomes of the species belonging to the genus *Bergenia* have been used in folk medicine for their antiscorbutic, astringent, diuretic, antipyretic, and ophthalmic properties [21]. The rhizomes are also used in dissolving kidney and gall bladder stones apart from healing cuts, burns, wounds, inflammation, cold, and cough [21]. The first use of the plant, “*Paashanbheda*”, for dissolving calculi and treatment of painful urination was narrated in ancient *Charaka Samhita* as early as 600 BC [5]. Ayurveda mentions the use of sap prepared from the leaves of *B. ligulata* for the treatment of urinary diseases, stomach problems, epilepsy, and cold [9]. Similarly, *Ayurveda* and *Unani* medicine systems also mention the utilization of *B. ligulata* roots in the management of vesicular calculi, urinary discharge, exorbitant uterine hemorrhage, ailments of the bladder, diarrhea, menorrhagia, excessive splenic growth, and cardiovascular diseases [7,22]. *Sushruta Samhita* mentions its use in the management of kidney and bladder stones and blood sugar. Some other indigenous Indian literature, including *Bhavaprakash*, *Rajnighantu,* and *Chakradatta,* also prescribed the use of *B. ligulata* in the management of urinary diseases and stones and the purification of the urinary bladder. Various local communities in the central Himalayan region consume different parts of the plant, such as the root rhizome, leaf, or whole plant in the form of sap or liquor or powder against dizziness, headache, vertigo, and kidney stones [9]. For example, the *Bhotia tribes* of central Himalayas consume dried rhizomes powder to treat kidney stones [23]. The tribal communities of Dharchula, Uttar Pradesh, India use roots of *B. ligulata* in healing cuts and wounds, ophthalmic problems, and dissolution of kidney stones and in urinary diseases [9]. The tribal communities in the Chamba district of Himachal Pradesh, India, use leaves of *B. ligulata* in the preparation of tea and consume it to treat common cold [24]. In the eastern Himalayas, leaves of this plant are used to treat cuts, wounds, and boils by the Monpa tribes of Arunachal Pradesh, India, whereas the Naga tribes of India use the roots in the management of liver diseases and tuberculosis [9]. Similarly, Mizo tribes in India use the decoction prepared from the roots of *B. ligulata* in the management of diarrhea and infection of pulmonary system. Juice prepared from the leaf is used against boils and is also taken orally to dissolve kidney stones [25]. *B. ligulata* is available as an over-the-counter herbal product, particularly as powder [26], as well as stems, roots, and seeds [27].

## 4. Bioactive Compounds in *Bergenia ligulata*

Ultra-high-performance liquid chromatography coupled with hybrid linear ion trap triple quadrupole mass spectrometry (UHPLC-QqQLIT-MS/MS) has been able to quantify eight major bioactive compounds from the rhizome of the plant: bergenin, arbutin, gallic acid, protocatechuic acid, chlorogenic acid, catechin, syringic acid, and ferulic acid [10]. In comparison with other medicinally important *Bergenia* species such as *B. ciliata, B. purpurascens,* and *B. stracheyi*, the highest total contents of these eight compounds have been noted in *B. ligulata* [10]. Other important phenolic compounds of *B. ligulata* include (+)-afzelechin [28,29], paashaanolactone [30], caryophyllene, 1,8-cineole, β-eudesmol, β-sitosterol, (+)-(6S)-parasorbic acid, 3-methyl-2-buten-1-ol, phytol, and tannic acid [31], isovalaric acid [32], stigmasterol [14], avicularin [7,33] terpinen-4-ol [34], quercetin [35], reynoutrin [36,37], and sitoinoside I [38]. These bioactive compounds, as presented in Table 1, are believed to be responsible for the medicinal properties of the plant [9].

### 4.1. Bergenin

Bergenin is a C-glucoside of 4-O-methyl gallic acid, a colorless crystalline polyphenol that comprises an aromatic ring, an annellated δ-lactone ring, and a glucopyranose ring [39]. It shows strong effectiveness in averting stress-induced gastric ulcers in rat models and is widely used as an ingredient of folk medicine for gastritis [40]. Cell line studies revealed that bergenin can induce apoptosis in HeLa (cervical cancer) cells by arresting the cell cycle at G0/G1 phase and can also promote anti-cancer activity by inhibiting the expression of signal transducer and activator of transcription 3 (STAT3) protein and metastasis of cancer cell [41]. Bergenin introduced the highest antioxidant and lipophilic properties which played a vital role in averting neuronal diseases and death [42]. It also accelerates the osteogenesis of bone mesenchymal stem cells through the upregulation of the sirtuin 1 (SIRT1) expression [43]. Strong antimicrobial activity of bergenin has been recorded against *Aspergillus flavus, Aspergillus niger, Escherichia coli, Enterococcus faecalis, Pseudomonas aeruginosa, Staphylococcus aureus,* and *Candida albicans* [44,45].

### 4.2. Catechins

Catechins are composed of two aromatic rings with hydroxyl group. This colorless crystalline polyphenol [46] has a molecular weight of 290 g/mol [47]. Depending on the distribution of hydroxyl group in aromatic ring, catechins are categorized into two types: (i) free catechins and (ii) esterified catechins [48]. Several studies show strong antiviral properties of catechins against adenovirus [49], enterovirus [50], human immunodeficiency viruses (HIV) [51], influenza virus [52], and tobacco mosaic virus (TMV) [53]. Catechins and their derivatives are effective scavengers of reactive oxygen species (ROS) [54]. Catechins moiety can function as free radical scavengers by requisitioning metal ions, and the B-ring site serves as a major site where the scavenging reaction is carried out [55,56,57]. Manikandan et al. (2012) reported that catechins possess significant anti-cancer properties and may reduce the proliferation of HCT 116, HCT 15 (human colon adenocarcinoma) and Hep G-2 (human larynx carcinoma) cell lines and are able to induce apoptosis [58].

### 4.3. Arbutin

Additionally known as *p*-hydroxyphenyl-β-d-glucopyranoside, arbutin is a bioactive hydrophilic polyphenol that has two isomers such as α-arbutin and β-arbutin [59]. Arbutin and its derivatives can directly control the overproduction of melanin by converting tyrosinase into L-DOPA (levodopa) and obstructing the tyrosinase activity without altering the mRNA expression [60]. Arbutin can introduce anti-inflammatory activity by decreasing the production of nitric oxide (NO) and expression of iNOS and cyclooxygenase-2 (COX-2) in lipopolysaccharide-stimulated BV2 cells (murine microglial cells) which also simultaneously suppressed the production of pro-inflammatory cytokines such as interleukin-1β (IL-1β) and tumor necrosis factor-α (TNF-α) and monocyte chemoattractant protein-1 (MCP-1) [61]. Li et al. (2011) reported that arbutin has antitumor activity by inducing TCCSUP (human bladder cancer) cell proliferation by inhibiting extracellular signal-regulated kinase (ERK) and by accelerating p21 protein expression [62].

### 4.4. Gallic Acid

Gallic acid is a secondary metabolite present in most plants and is also known as 3,4,5 trihydroxybenzoic acid [63]. Gallic acid alters the integrity of bacterial cell membranes by penetrating the bacterial cell wall and disrupting the cellular respiration and electron transport chain. This compound also negatively alters DNA cleavage by affecting dihydrofolate reductase activity in bacteria [64]. It also shows antiviral activity with respect to hepatitis C virus (HCV) [65] and herpes simplex virus (HSV) [66]. Gallic acid showed anti-cancer activity by arresting the cell cycle and promoted apoptosis by activating the caspases pathway and can also reduce metastasis [64]. Gallic acids also possess gastroprotective activity [67], anti-hypertriglyceridemia activity, and diet-induced anti-hyperglycemic activity [68].

### 4.5. Protocatechuic Acid

Protocatechuic acid is a secondary metabolite that consists of an aromatic ring and one or more hydroxyl groups and is also known as 3,4-dihydroxybenzoic acid [69]. Protocatechuic acid possesses free radical scavenging property and shows antioxidant activity through reduction of ROS generation in different parts of the body such as the brain, heart, kidney, and liver, and prevents different degenerative diseases [70]. Protocatechuic acid reportedly shows preventive activity against neurodegenerative diseases such as Alzheimer’s and Parkinson’s diseases by interrupting the aggregation of β-amyloid plaques in brain tissues and preventing hyperphosphorylation of tau protein in neurons [71]. It has the ability to inhibit the growth of Gram-positive and Gram-negative bacteria and also shows antifungal activity [70]. In addition to antioxidant and anti-inflammatory activities, protocatechuic acid produced a significant positive effect in hyperglycemic conditions by increasing plasma insulin level [72]. Lende et al. (2011) suggested that protocatechuic acid can introduce promising anti-inflammatory properties by reducing carrageenan-induced paw edema and Freund’s adjuvant arthritis [73].

### 4.6. Chlorogenic acid

Chlorogenic acid is a phenolic secondary metabolite and is also known as 5-O-caffeoylquinic acid (5-CQA). Chlorogenic acid plays a role in glucose metabolism through the activation of AMP-activated protein kinase (AMPK), which leads to glucose transport from intracellular membrane to plasma membrane by glucose transporter-4 (GLUT4) and increases cardiac glycolysis by activating phosphofructokinase 2 [74]. It can inhibit glucose-6-phosphatase activity and reduce glucose level in circulation, which causes less deposition of fatty acids in the adipose tissue and simultaneously utilizes the stored fat in the body, resulting in a reduction in body weight. It also shows anti-diabetic properties [75]. In addition to antioxidant activity, chlorogenic acid also possesses antibacterial activity against *Escherichia coli, Klebsiella pneumonia, Staphylococcus epidermidis* and antifungal activity against *Candida albicans* and antiviral activities against HIV, HSV-1, and HSV-2 and adenovirus [75].

### 4.7. Syringic Acid and Ferulic Acid

Syringic acid and ferulic acid are phenolic compounds. Syringic acid contains methoxy groups on the aromatic ring at positions of 3 and 5 [76]. Ferulic acid is also known as 4-hydroxy-3-methoxycinnamic acid [77,78]. Ferulic acid is a potent photoprotective and brightening agent for skin care; it nourishes the skin by balancing collagen and elastin activity [79]. Ferulic acid is also a potent scavenger of free radicals, and it can decrease lipid peroxidation rate in the rat brain by donating electrons from hydroxy and phenoxy groups to neutralize ROS [80]. Ferulic acid has also demonstrated antimicrobial activities against *Enterococcus faecalis*, *Staphylococcus aureus* [81], and *Cronobacter sakazakii* [82]. Treating *Cronobacter sakazakii* infection with syringic acid deteriorated bacterial cell membrane structure and halted bacterial growth, and this compound can be used as a natural preservative, too [82]. In addition to antioxidant activity, syringic acid appeared effective against acute pancreatitis [83], renal ischemia-reperfusion injury [84], and demyelination and inflammation in sciatic nerves [85]. Syringic acid enhances the working capacity of *β*-cells in the pancreas and increases the plasma insulin level, which induces more deposition of glycogen in peripheral tissue [85,86].

## 5. Potential Clinical Use

*Bergenia* species have been well known since ancient times for their potential curative effects on different human ailments [37]. *B. ligulata* is one of the important members of this genus and, also shows an extensive range of pharmacological activities. It is widely used in Indian traditional medicine as well as other traditional medicine systems of the world. The bioactive compounds found in the plant are diverse and may be responsible for the pharmacological activity of the plant. In traditional medicine systems, the plant is used mainly for its antilithiatic activity; additionally, the whole plant or parts of the plant (root and rhizome) have been used in the management of fever, inflammation, diabetes, microbial infections, wounds, burns [21], amelioration of liver disease, urinary crux, and abdominal and heart diseases [7].

### 5.1. Antilithiatic Activity

In Indian traditional medicine, the rhizomes of *B. ligulata* have been considered a potential drug in the management of renal stones [87]. Dichloromethane, a bioactive fraction of *B. ligulata* extract showed high efficiency against kidney stone aggregation when administered orally for 21 days at a dose of 7 mg/kg body weight [88]. Spectroscopic analysis revealed bergenin to be the potent antilithiatic bioactive molecule isolated from rhizomes of *B. ligulata* [89]. Methanolic extract of *B. ligulata* and bergenin exhibited marked dissolution of urinary calculi both in kidney and urine constituents [90]. Treatment of rats with aqueous-methanolic extract of *B. ligulata* rhizomes at a dose range of 5–10 mg/kg body weight for 21 days was able to prevent ethylene glycol-induced urolithiasis by inhibiting CaC_2_O_4_ crystal deposition in the renal tubules and simultaneously improved renal function [15]. An in vitro study confirmed that supplementation of dried leaves aqueous extract of *B. ligulata* has strong inhibitory potential against calcium oxalate monohydrate (COM) and hydrogen phosphate dehydrate crystal formation [91,92]. In ethylene glycol-induced hyperoxaluric rats, the dysfunction of mitochondria during stone crystal formation was manifested by reducing activities of electron transport chain complexes I, II and IV and, also by increasing mitochondrial oxidative stress. Oral administration of bergenin at a dose of 10 mg/kg for 28 days in ethylene glycol-induced hyperoxaluric rats showed amelioration of the damages to mitochondrial complexes as well as the alleviation of oxidative stress showing its potential effectiveness against urolithiasis [93]. A recent study on renal epithelial NRK-52E cells showed that ethanolic extract of *B. ligulata* significantly inhibits the nucleation and aggregation process of calcium oxalate crystals, further modulating the crystal structure by converting COM to the less pernicious form of calcium oxalate dihydrate (COD) [16].

### 5.2. Antipyretic Activity

Pyrexia or fever may occur due to acute infection or inflammation, or even due to any injury to tissue leading to the release of cytokines that initiate the synthesis of prostaglandin E2 (PgE2) in the hypothalamic area [94]. *B. ligulata* is considered an antipyretic herbal drug, and particularly the dried rhizome is used to prevent such fever [9]. Another study showed that ethanolic extract of roots and rhizomes of *B. ligulata* exhibit antipyretic activity at a dose of 500 mg/kg body weight in albino Wistar rats against yeast-induced fever. The rectal temperatures were recorded at time intervals of 1, 2, 3, 4 and 5 h after administration of *B. ligulata* extract [95].

### 5.3. Anti-Diabetic Activity

Plants belonging to the genus *Bergenia* play a crucial role in reducing the hyperglycemic condition. Rigorous studies on animal models revealed that *B. ligulata* possesses strong anti-diabetic activity [37]. Another possible mechanism of anti-diabetic action of *B. ligulata* may be attributed to its bioactive compound (+)-afzelechin, which acts as an inhibitor of α-glucosidase enzyme, as ascertained by enzyme inhibition assay [9,96]. The inhibition of α-glucosidase enzyme has been found to be effective in the treatment of hyperglycemia by delaying the absorption of carbohydrates in rat small intestines [96].

### 5.4. Anti-Inflammatory Activity

Bergenia species have strong anti-inflammatory activity as both the aqueous as well as ethanolic extracts of rhizomes showed effective anti-inflammatory potential in rat models [37]. *B. ligulata* is a well-known herb among folklore medical practitioners due to its anti-inflammatory potential. A study regarding the anti-inflammatory activity ensured its bioactive effect, where the oral application of aqueous, as well 50% ethanolic extracts of *B. ligulata* at a dose of 1 g/kg body weight of male Wistar rats, was able to attenuate the inflammatory response by reducing the level of succinate dehydrogenase (SDH), a key enzyme the level of which has been reported to rise during inflammation [6]. A possible explanation behind the therapeutic effect may be attributed to the bioactive molecule bergenin [97]. A new study on NRK-52 E cells revealed the reduction in inflammatory mediators such as MAPK, OPN, and nuclear factor kappa B (NF-ĸB) in presence of *B. ligulata* extract [16].

### 5.5. Hepatoprotective Activity

*Bergenia* species also have a hepato-protective effect. The administration of ethanolic root extract of *B. ligulata* to albino Wistar rats at a dose range of 25–35 g/kg body weight for 10 days exerted hepatoprotective activity, which was assessed by measuring the levels of serum glutamate pyruvate transaminase (SGPT), serum glutamate oxaloacetate transaminase (SGOT), serum alkaline phosphatase (ALP), and total bilirubin. All these parameters were significantly lower in the *B. ligulata*-treated group as compared to standard drugs [95].

### 5.6. Cardioprotective Activity

The cardioprotective potential of *B. ligulata* is also attributed to its phytochemical constituents. Several studies have highlighted its effects on different mammalian models. The administration of *B. ligulata* extract at a dose of 50 mg/kg body weight in dogs through an intravenous route showed effective hypotensive activity [7,98]. Alcoholic extract of rhizome also exhibited anti-bradykinin action without altering the action of 5-hydroxytryptamine (5-HT) receptor and acetylcholine as reported from isolated guinea pig ileum [7,9]. The scientific experiments that are carried out in mammalian models shows various pharmacological activities of the *B. ligulata* (Table 2). However, further studies are required for validation of these activities of the bioactive molecules present in the herb.

## 6. Possible Mechanism(s) of Action

Bioactive molecules of *B. ligulata* include polyphenols, flavonoids, and quinones that contribute to the pharmacological properties of the plant. The major ingredients of polyphenols include bergenin, arbutin, and catechin, which mostly add value to the medicinal value of the plant [37,100].

### 6.1. Anti-Urolithiatic Mechanism

**Figure 2:**
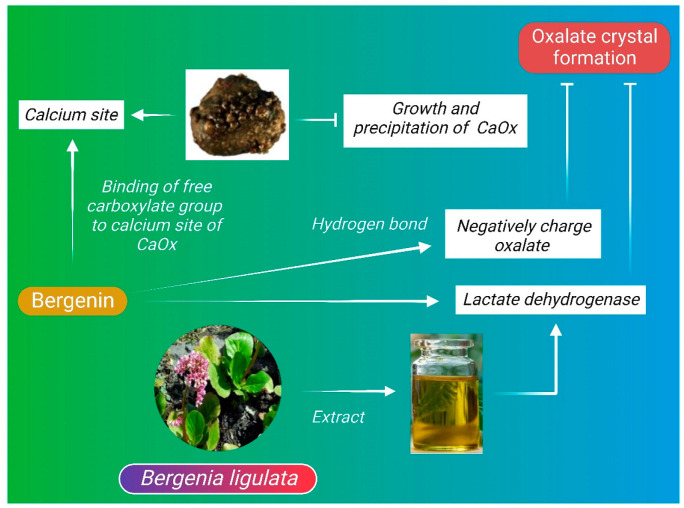
Anti-urolithiatic mechanism of *B. ligulata*. Bergenin, one of the bioactive components of *B. ligulata,* makes it a potent anti-lithiatic herb. Hydrolysis product of bergenin, i.e., 4-O-methylglycoside (4-OMG), has negatively charged free carboxylate group, which enables it to bind with the calcium site of calcium oxalate (CaOx) crystals and inhibit the precipitation of these crystals, thereby impeding renal stone formation.

The composition of kidney stones mainly depends on the physiologically and chemically altered urine. Depending on the chemical deposition, kidney stones can be classified as calcium stones—i.e., stones formed due to aggregation of calcium oxalate (CaOx) and calcium phosphate (Ca_3_(PO_4_)_2_) in renal calculi [101]. Other forms of stone include magnesium ammonium phosphate stones; uric acid stones; cystine stones; and drug-induced stones [101]. The general process of stone formation is common for all types of kidney stones. The most common, i.e., the calcium stone formation process, is mainly attributed to the crystallization of calcium oxalate, and the process involves nucleation, crystal development, aggregation, and the retention of crystal in the renal duct [93,101]. The wide range of action of bergenin can be demonstrated by its inhibitory effect on formation of kidney stones. Bergenin is a C-glycoside of 4-O-methyl gallic acid with an ionizing ability in buffer medium under neutral pH, whereas 4-O-methylglycoside (4-OMG) [102], the hydrolysis product of bergenin has negatively charged free carboxylate group, which enables it to bind with the calcium site of CaOx crystals and inhibit the growth and precipitation of CaOx crystals [89]. Similarly, other phenolic groups of *B. ligulata* such as arbutin and catechin also has the ability to interact with negatively charged oxalate ions by hydrogen bonds, which ultimately modulates the growth of CaOx crystals. Treatment with *B. ligulata* extract directly reduces the activity of lactate dehydrogenase (Figure 2), which is required for the formation of oxalate crystals in kidney [89].

### 6.2. Antioxidative Mechanism

**Figure 3:**
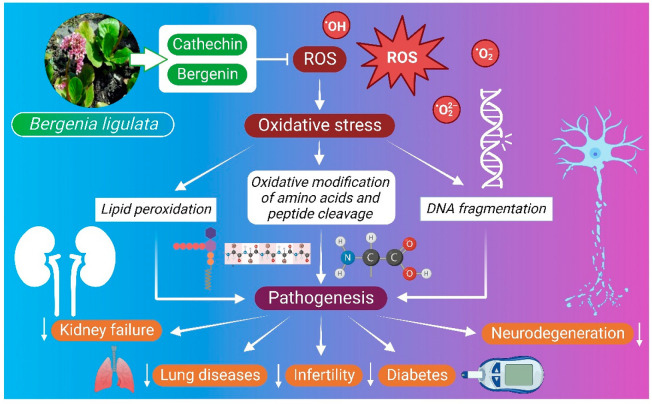
Antioxidative mechanism of *B. ligulata***.** Bergenin has been attributed to the free radical scavenging capacity against hydroxyl radicals through the formation of aromatic conjugated dienes and Fe (II) complex, which ultimately block the generation of hydroxyl radicals. One of the compounds of *B. ligulata,* 11-O-galloylbergenin, has a special structural orientation due to the presence of two meta and one para group, which induces free radical scavenging by the herb. ROS—reactive oxygen species.

Biological reactions in living organisms produce unpaired-electron-containing molecules called free radicals, referred to as ROS and reactive nitrogen species (RNS). Antioxidants are the main defensive mechanism that neutralizes the action of free radicals. A balance between ROS and antioxidants is crucial for maintaining normal biological activity in living organisms. A minimal amount of free radicals are essential for immune responses, phagocytosis, activation of cellular receptors, processing of cellular signaling, and other important biological activities [103], whereas an excessive amount of free radical generation makes cells susceptible to oxidative stress, which ultimately promotes lipid peroxidation, DNA damage, oxidative modification of amino acid, and oxidative-stress-mediated peptide cleavage [103,104]. Bergenin and catechin are two major compounds found in *B. ligulata* that may mostly contribute to the antioxidant property of the plant [14]. Bergenin introduces free-radical-scavenging capacity against hydroxyl radicals by forming aromatic conjugated dienes, and it can also form a complex with Fe (II) that ultimately blocks the generation of hydroxyl radicals in the Fenton reaction [89]. The superoxide anion radical scavenging property of bergenin was assessed through the NADH radical scavenging assay, and the reducing property of the bioactive molecule was attributed to its electron-shifting ability [105]. The 11-O-galloylbergenin of *B. ligulata* has a benzoyl moiety with three hydroxyl groups, i.e., two meta and one para group, which provides 11-O-galloylbergenin a special structural orientation to easily interact with free radicals and scavenge on them [11] (Figure 3).

### 6.3. Antipyretic Mechanism

**Figure 4:**
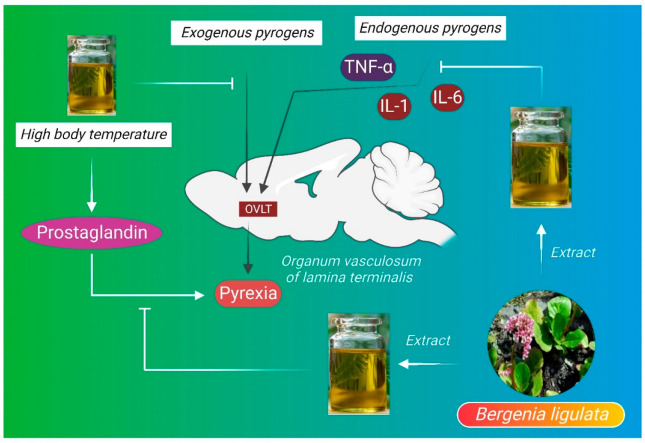
Antipyretic mechanism of *B. ligulata*. The biomolecules present in *B. ligulata* have been suggested to block the activity of pyrogens on temperature-sensitive neurons in the preoptic area of hypothalamus, thereby ameliorating fever. IL-1—interleukin-1, IL-6—interleukin-6, OVLT—organum vasculosum laminae terminalis.

Pyrexia is defined as the abnormal elevation of body temperature, where external temperature is more than 38 °C or internal temperature is above 38.4 °C [106]. In the case of pyrogenic pyrexia, the generation of fever involves several mechanisms, most commonly exogenous pyrogens such as microbes or endogenous pyrogens such as interleukin 1 (IL-1) or interleukin 6 (IL-6). TNF-α interacts with organum vasculosum of the lamina terminalis (OVLT), leading to the development of pyrexia. On the contrary, most autoinflammatory conditions are genetic and also related to malfunctioning of pro-inflammatory cytokines such as interlukin-1 or interferon signaling or constitutive NF-kB (nuclear factor kappa-light-chain-enhancer of activated B cells) activation, which are the most potent targets for the management of pyrexia [107]. In pyrexic conditions, there is an increase in prostaglandins (PGE2), which control the thermoregulatory center in the hypothalamus. Some antipyretic drugs introduce their antipyretic activity by blocking prostaglandin biosynthesis [108]. *B. ligulata* has antipyretic properties, particularly ethanolic root and rhizome extracts of the plant contain steroids, alkaloids, flavonoids, and terpenoids, which may block the activity of pyrogens on temperature-sensitive neurons in the preoptic part of the hypothalamus [17]. However, more detailed study is needed to reveal the bioactive molecules that are responsible for the antipyretic property (Figure 4).

### 6.4. Anti-Diabetic Mechanism

**Figure 5:**
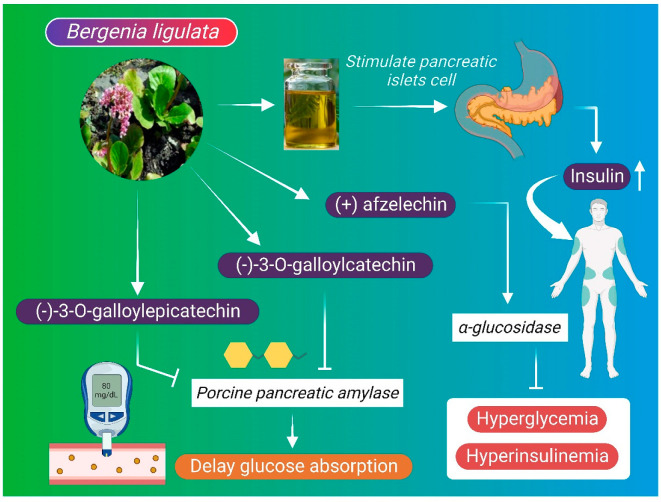
Anti-diabetic mechanism of *B. ligulata*. Plant extract stimulates the pancreatic islet cells and increases insulin secretion for maintaining normal blood glucose levels. (+) afzelechin, a compound of *B. ligulata*, inhibits the enzymatic action of alpha-glucosidase, thus delaying the absorption of dietary carbohydrate in the small intestine and reduces postprandial hyperglycemia and hyper-insulinemia. Additionally, compounds such as (-)-3-O-galloylepicatechin and (-)-3-O-galloylcatechin from *B. ligulata* inhibit pancreatic α-amylase and delay the absorption of glucose in the intestine.

Diabetes mellitus has several etiologies and is characterized by high blood glucose level resulting from the destruction of pancreatic beta cells, defects in insulin secretion, and abnormalities in insulin receptors [109]. Diabetes can also trigger other diseases such as cardiovascular diseases, neuropathy, retinopathy, and nephropathy [96]. *B. ligulata* extract may stimulate the pancreatic islet cells and increase insulin secretion to maintain normal blood glucose levels [7]. Afzelechin from *B. ligulata* extract exhibited anti-diabetic activity by inhibiting the enzymatic action of α-glucosidase, thus delaying the absorption of dietary carbohydrates in the small intestine and reducing postprandial hyperglycemia and hyper-insulinemia [96,110]. Similarly, (-)-3-O-galloylepicatechin and (-)-3-O-galloylcatechin isolated from *B. ligulata* have demonstrated inhibitory effects against porcine pancreatic α–amylase (Figure 5), which also delays the absorption of glucose in the intestine [9,111].

### 6.5. Anti-Inflammatory Mechanism

**Figure 6:**
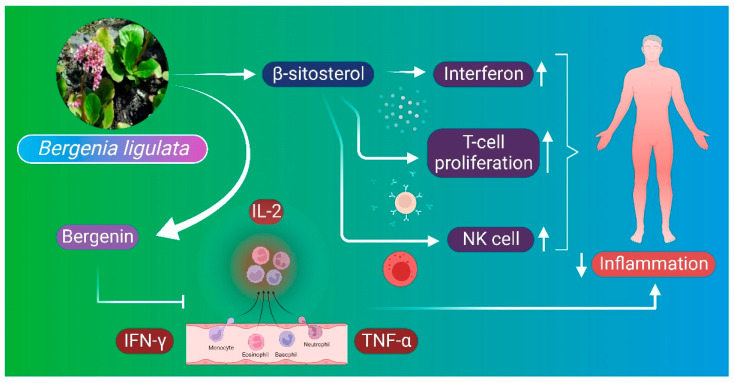
Anti-inflammatory mechanism of *B. ligulata*. Beta-sitosterol is an important constituent of *B. ligulata* plant extract that possesses anti-inflammatory properties. It induces the proliferation of T cells and releases interferon. Anti-inflammatory properties of the plant are attributed to the synergistic effect of pro-inflammatory enzyme inhibitors that are responsible for reducing the inflammatory response. Bergenin also inhibits the formation of proinflammatory cytokines such as interleukin-2 (IL-2), interferon gamma (IFN-ɣ), and tumor necrosis-alpha (TNF-α), all assisting in the anti-inflammatory response. NK—natural killer cell.

Inflammation is a response of the immune system to foreign substances and injuries, which is essential for tissue homeostasis under different noxious conditions [112]. Beta-sitosterol is an important constituent isolated from *B. ligulata* extract that possesses anti-inflammatory properties. Beta-sitosterol directly induces the proliferation rate of T cells and releases interferon and increases natural killer cell activity as well. Anti-inflammatory properties of the *B. ligulata* plant may also be attributed to the synergistic effect of pro-inflammatory enzyme inhibitors that are responsible for the reduction in inflammatory response [6]. Bergenin also inhibits the formation of proinflammatory cytokines such as IL-2, interferon gamma (INF-ɣ), and TNF–α [45]. It also shows anti-microbial properties by inhibiting the growth of microbes in both in vivo and in vitro (Figure 6); however, the exact mechanism of action is yet unknown [37].

### 6.6. Hepatoprotective Mechanism

**Figure 7:**
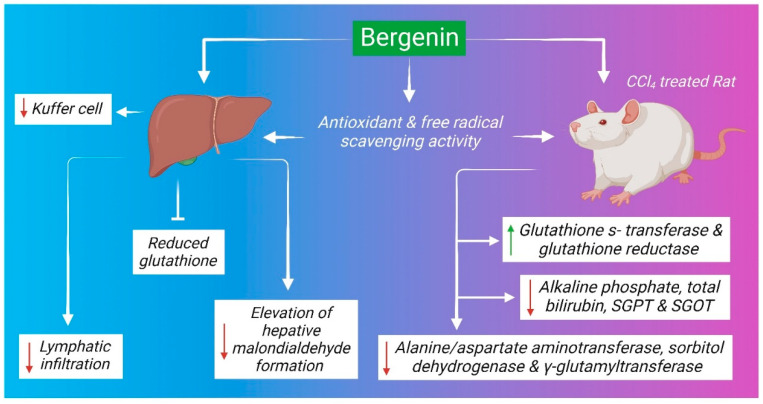
Hepatoprotective mechanism of *B. ligulata*. The plant extract shows hepatoprotective properties through antioxidant activity and free radical scavenging activity. Bioactive compounds reduce the number of Kuffer cells in the liver and inhibit the infiltration of lymphocytes. It also reduces alkaline phosphate, total bilirubin, serum glutamic-pyruvic transaminase (SGPT) and serum glumatic-oxaloacetic transaminase (SGOT), alanine aminotransferase (ALT), sorbitol dehydrogenase, and γ-glutamyl transferase. Further, it helped resist the depletion of reduced glutathione content and inhibit malondialdehyde formation in the liver of CCl_4_-treated rats.

Several studies have demonstrated the hepatoprotective activity of *B. ligulata*, although the mechanism of action is poorly understood. The antioxidant properties and cellular restoration capacity of the plant may play significant roles in the recovery of damaged liver tissues. Free radicals such as hydroxyl radicals, hydrogen peroxide, superoxide radicals, and lipid peroxide are predominant in liver diseases. These free radicals are normally generated during the biochemical process of the body or due to exposure to different environmental toxicants or pathological states [113]. Excess amounts of free radicals generate oxidative stress that alters the membrane structure and damage other important components of the cell including lipids, proteins, and nucleic acids [114]. The bioactive compounds of *B. ligulata* may exert hepatoprotective activity (Figure 7) through antioxidant and free-radical scavenging properties. Similarly, it can normalize increased Kuffer cells number and lymphocytic infiltration in infected mice [19]. In Wistar albino rats, *B. ligulata* root extract ameliorated carbon tetrachloride (CCl_4_)-induced liver damage along with a reduction in the level of alkaline phosphatase (ALP), total bilirubin level, SGPT, and SGOT [95]. The major bioactive component of *B. ligulata*, i.e., bergenin, might be responsible for the hepatoprotective properties of the plant. Bergenin at doses of 50, 100, and 200 mg/kg body weight showed a strong hepatoprotective effect when administrated orally for 7 successive days in CCl_4_-induced liver damage in rats. The administration of bergenin subsequently normalizes the increasing serum enzymatic activities of alanine/aspartate aminotransferase, sorbitol dehydrogenase, and γ-glutamyltransferase in CCl_4_-treated rats in a dose-dependent manner. In contrast, the recuperation of the activities of glutathione S-transferase and glutathione reductase was also reported. Additionally, bergenin can prevent the elevation of hepatic malondialdehyde formation and the depletion of reduced glutathione content in the liver of CCl_4_-intoxicated rats [115,116].

## 7. Future Perspectives

Plant-based natural remedies have been used worldwide for the management of various human ailments for ages. In developing countries, approximately 8 out of 10 people opt for herbal treatment for their primary health care, due to easy accessibility, low toxicity, and inherited traditional knowledge of using plant and their derivatives in the form of either the whole plant or part of the plant, such as root, rhizome, leaf, fruit, or flower. In this modern era, researchers are focusing on herbal medicines by taking reference from indigenous ancient traditional literature such as *Ayurveda*, *Unani*, *Siddha,* traditional Chinese medicine, and others, as a reverse pharmacology approach. Cutting-edge analytical tools and techniques are paving the way for the identification and validation of bioactive molecules from herbs and their use on par with standard modern synthetic drugs. The bioactive molecules of various traditionally used plants have been clinically proven as effective against a range of acute and chronic diseases. Further studies on the traditional herbs may lead to development of several novel drugs against various long-standing human ailments. In this review, we attempted to provide the mechanism(s) of action of *B. ligulata* and/or its phytoconstituents to better understand how the plant executes its anti-urolithiatic, antioxidative, antipyretic, anti-diabetic, anti-inflammatory, and hepatoprotective properties, as well as discuss the important bioactive compounds of clinical importance. The studies conducted on rats and other mammalian models provide a deep insight into the therapeutic potential of *B. ligulata* in the management of various ailments. Despite limited data, *B. ligulata* has been shown to exert antioxidant properties, reduce cellular oxidative stress, and modulate enzyme action (α-glucosidase) under in vivo and in vitro conditions, which renders the herb interesting for further clinical studies.

## Figures and Tables

**Table 1:** Bioactive molecules found in *Bergenia ligulata*.

Chemical Structure	Name of the Molecule	Reference(s)
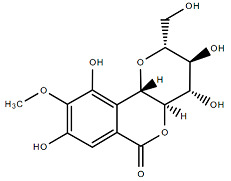	Bergenin	[10]
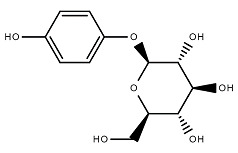	Arbutin	[10]
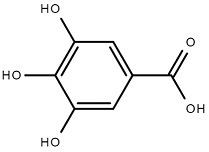	Gallic acid	[10]
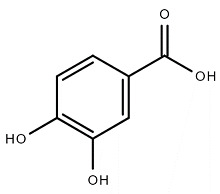	Protocatechuic acid	[10]
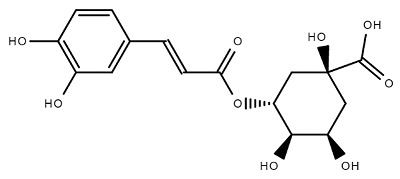	Chlorogenic acid	[10]
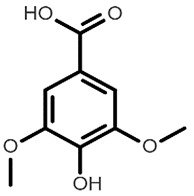	Syringic acid	[10]
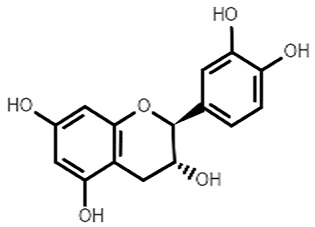	Catechin	[10]
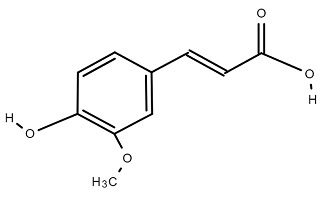	Ferulic acid	[10]
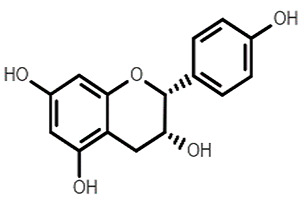	(+)-afzelechin	[28,29]
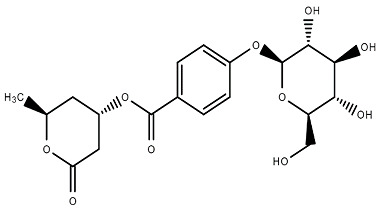	Paashaanolactone	[30]
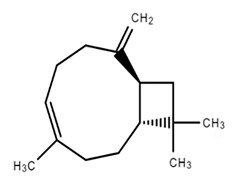	Caryophyllene	[31]
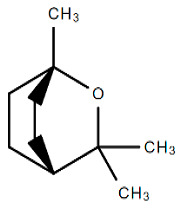	1,8-cineole	[31]
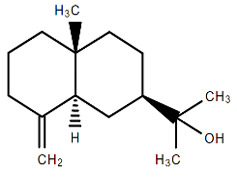	β-eudesmol	[31]
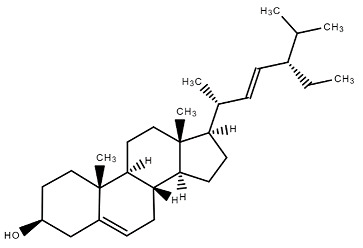	Stigmesterol	[14]
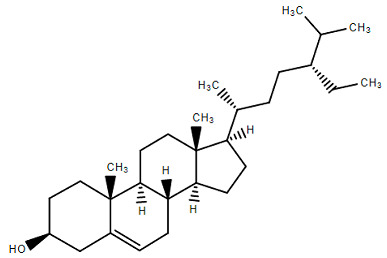	β-sitosterol	[31]
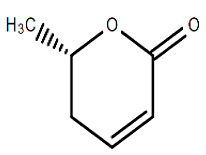	(+)-(6S)-parasorbic acid	[31]
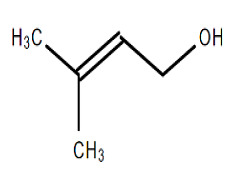	3-methyl-2-buten-1-ol	[31]
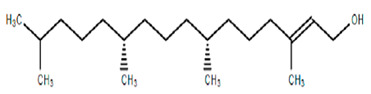	Phytol	[31]
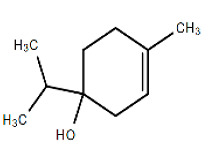	Terpinen-4-ol	[34]
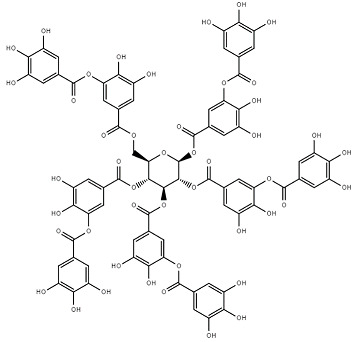	Tannic acid	[31]
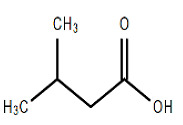	Isovalaric acid	[32]
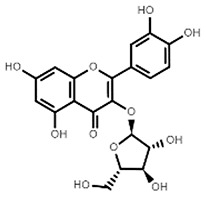	Avicularin	[7,33]
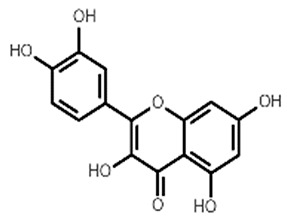	Quercetin	[35]
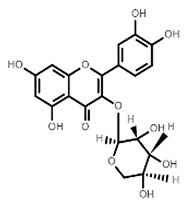	Reynoutrin	[31,37]
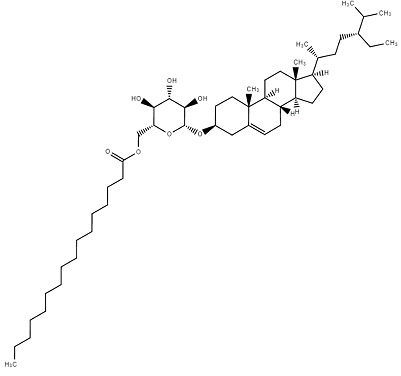	Sitoinoside I	[38]

**Table 2:** Clinical significance of *Bergenia ligulata* in various experimental models, and their key findings along with calculated human doses.

Plant Part/Chemical Constituent	Mode of Study	Experimental Model	Study Design	Dosage	Calculated Human Dose * [99]	Action	Reference(s)
Ethanolic extract of roots and rhizomes of *B. ligulata*	In vivo	Wistar rat	Yeast induced fever	500 mg/kg	81.08 mg/kg	Rectal temperatures were recorded at a time interval of 1, 2, 3, 4, and 5 h after administration and reduction in temperate was recorded	[17]
Aqueous-methanolic extract of *B. ligulata* rhizomes	In vivo	Rat	Ethylene glycol-induced urolithiasis	5–10 mg/kg	1.62 mg/kg	Inhibition of calcium oxalate (CaC_2_O_4_) crystal deposition in the renal tubules and simultaneous improvement of renal function	[15]
Bergenin	In vivo	Rat	Ethylene glycol-induced hyperoxaluria	10 mg/kg	1.62 mg/kg	Amelioration of damages to mitochondrial complexes as well as the alleviation of oxidative stress	[93]
Dichloromethane bioactive fraction of *B. ligulata*	In vivo	Rat	Ethylene glycol-induced renal calculi	7 mg/kg	1.13 mg/kg	Inhibition of kidney stone aggregation	[88]
Aqueous and 50% ethanolic extracts of *B. ligulata*	In vivo	Wistar rat	Orally administered	1 g/kg	0.16 gm/kg	Reduction in inflammatory response by lowering the SDH level	[6]
Ethanolic root extract of *B. ligulata*	In vivo	Wistar rats	Orally administered	25–35 gm/kg	5.67 gm/kg	Showed promising hepatoprotective effect by lowering the SGOT, SGPT and ALP levels	[95]
*B. ligulata* extract	In vivo	Dog	Orally administered	50 mg/kg	27.02 mg/kg	Promising hypotensive activity observed	[7,98]

* Human dose calculation: Reagan-Shaw et al. (2008) formulated conversion of animal doses to human equivalent dose (HED) based on body surface area (BSA). HED (mg/kg) = Animal dose (mg/kg) X animal K_m_ factor/human K_m_ factor. K_m_ is calculated by dividing body weight (kg) by BSA (m^2^). Considering 60 kg, 10 kg, and 0.15 kg body weight and 1.6, 0.5 and 0.025 BSA (m^2^) for human, dog, and rat, respectively.

## Data Availability

The study did not report any data.
